# Labor Induction with Orally Administrated Misoprostol: A Retrospective Cohort Study

**DOI:** 10.1155/2017/6840592

**Published:** 2017-09-18

**Authors:** Tove Wallstrom, Hans Jarnbert-Pettersson, David Stenson, Helena Akerud, Elisabeth Darj, Kristina Gemzell-Danielsson, Eva Wiberg-Itzel

**Affiliations:** ^1^Department of Clinical Science and Education, Karolinska Institutet, Women's Clinic, Sodersjukhuset, Sweden; ^2^Department of Immunology, Genetics and Pathology, Uppsala University, Uppsala, Sweden; ^3^Department of Public Health and Nursing, NTNU, Norwegian University of Science and Technology, Trondheim, Norway; ^4^Department of Women's and Children's Health, Karolinska Institutet, Karolinska University Hospital, Stockholm, Sweden

## Abstract

**Introduction:**

One great challenge in obstetric care is labor inductions. Misoprostol has advantages in being cheap and stable at room temperature and available in resource-poor settings.

**Material and Methods:**

Retrospective cohort study of 4002 singleton pregnancies with a gestational age ≥34 w at Sodersjukhuset, Stockholm, during 2009-2010 and 2012-2013. Previously used methods of labor induction were compared with misoprostol given as a solution to drink, every second hour. Main outcome is as follows: Cesarean Section (CS) rate, acid-base status in cord blood, Apgar score < 7,5′, active time of labor, and blood loss > 1500 ml (PPH).

**Results:**

The proportion of CS decreased from 26% to 17% when orally given solution of misoprostol was introduced at the clinic (*p* < 0.001). No significant difference in the frequency of low Apgar score (*p* = 0.3), low aPh in cord blood (*p* = 0.1), or PPH (*p* = 0.4) between the different methods of induction was studied. After adjustment for different risk factor for CS the only method of induction which was associated with CS was dinoproston^⁎⁎^ (Propess®) (aor = 2.9 (1.6–5.2)).

**Conclusion:**

Induction of labor with misoprostol, given as an oral solution to drink every second hour, gives a low rate of CS, without affecting maternal or fetal outcome.

## 1. Introduction

Even today, hundreds of thousands of women will die or suffer high levels of morbidity because of complications related to delivery [1]. Many of these deaths are avoidable. One of the greatest challenges in obstetric care is induction of labor.

In 2011, labor was induced in 15–20% of all singleton pregnancies in Sweden [2–7]. Most common methods of induction are amniotomy, mechanical dilatation with a balloon catheter, pharmacological inductions with prostaglandin E1 (misoprostol), prostaglandin E2 (dinoproston), or oxytocin.

High risks of both maternal as well as fetal complications are related to induction of labor. Recent published data of expectant management versus induction of labor shows that there is a significantly elevated risk for Cesarean Section (CS) in full time induced pregnancies, even after controlling for suspected confounders [8–11].

Misoprostol is a prostaglandin E1 analogue, developed for the treatment and prevention of gastric ulcers [11, 12]. Its proven efficacy of uterine contractility and cervical ripening has led to a drug currently being used for termination of unwanted pregnancy, management of incomplete and spontaneous abortions, induction of labor, augmentation of labor, and treatment of Postpartum Hemorrhage (PPH) [11]. Misoprostol has advantages in being cheap and stable at room temperature and widely available also in most resource-poor settings. Misoprostol is included in the World Health Organization (WHO) essential medicine list on several indications including labor induction [11]. Orally administrated misoprostol has in several publications been shown to be an effective method for induction of labor, comparable with PGE2 and oxytocin [11, 13, 14].

Due to high numbers of CS among induced pregnancies at the clinic of Sodersjukhuset, Stockholm, Sweden, a decision of changing the method for labor induction was taken in 2011. Senior consultants had experience from other settings where an orally administrated solution of misoprostol had been used, and a similar method was chosen and introduced at the clinic. The aim of this study was to compare previously used methods of labor induction with an orally administrated solution of misoprostol according to defined outcomes of deliveries.

## 2. Material and Methods

A retrospective study of labor induction was performed at the Department of Obstetrics at Sodersjukhuset, Stockholm, Sweden. Sodersjukhuset is a large delivery ward, with about 7,500 deliveries per year. The frequency of labor inductions was about 16% during study time. Primary endpoint of the study was whether a CS was performed or not. Secondary endpoints were acid-base status in cord blood at delivery, Apgar score < 7 after 5 min, active time of labor, and PPH (blood loss > 1500 ml) among induced deliveries.

Previously, different types of medical preparations such as vaginal prostaglandin E2 (Minprostin®, a vaginal gel, Pfizer, NY, USA, or Propess, an insert with slow-release of PGE2, Ferring, Malmo, Sweden) or mechanical methods (balloon catheters, Bard®/Rusch®, Cook Medical, USA) were used at the clinic as primary methods for labor induction in the case of an immature cervix (Bishop score (BS) ≤ 5). Late in 2011, the method of induction was changed at the clinic, from vaginal PGE2 to the use of an orally administrated solution of misoprostol as first-line option. Data from all induced deliveries taking place at the clinic during the years of 2009, 2010, 2012, and 2013 were collected from the database of “Obstetrix.” The year of 2011 was excluded as the primary method of induction at the clinic was changed during that year.

Inclusion criteria for the study were women with a fetus in cephalic presentation, singleton, and induction of labor at gestational age ≥34 weeks. Exclusion criteria were women with noncephalic presentation, multiples, fetal malformations, Intrauterine Fetal Death (IUFD), or prematurity <34 weeks of gestation. Methods of induction, pregnancy baseline data, and delivery outcomes were compared among all the included deliveries. All personal data were encoded, so that individuals could not be identified in the analysis.

After inclusion, deliveries were divided into six groups according to the method of induction, misoprostol, dinoproston^*∗*^ (Minprostin), dinoproston^*∗∗*^ (Propess), balloon catheter (Bard), amniotomy, or oxytocin administrated primarily for augmentation.

To prepare the misoprostol oral solution, one tablet of 200 *μ*g misoprostol was dissolved in 20 ml of water. The solution thus held 10 *μ*g of misoprostol/ml. This method of administration has been tested by the Swedish Institute of Pharmacology [15] and approved to be accurate in terms of correct dosage. A dose of 2.5 ml/25 *μ*g of misoprostol was administered orally to the women every two hours until frequent painful contractions were obtained. The dose could be repeated up to eight times if necessary. When ripening of the cervix had been achieved (BS ≥ 5), amniotomy and oxytocin were used to support uterine contractions.

In the dinoproston^*∗*^ (Minprostin) group, 1 or 2 mg of dinoproston was given vaginally. The progress of induction was evaluated by a vaginal examination every six hours. In the case of a still immature cervix, additional doses were administered, up to a total of three doses (6 mg). When ripening of the cervix had been achieved (BS ≥ 5), amniotomy and oxytocin were used to support labor contractions.

In the dinoproston^*∗∗*^ (Propess), group, a slow-release insert was inserted vaginally. The insert could remain in place for up to 24 hours, or until contractions were achieved. The progress of induction was evaluated by a vaginal examination. When ripening of the cervix had been achieved (BS ≥ 5), amniotomy and oxytocin were used to support contractions.

In the balloon group, mechanical induction was performed with a balloon catheter (Bard). The balloon was introduced into the cervix beyond the internal os and the bulb inflated with 50 ml of sterile water. At a cervical dilation of at least 3 cm, the balloon falls out and the induction was then followed by amniotomy and oxytocin.

Amniotomy followed by stimulation with oxytocin was used in deliveries with a mature cervix, and oxytocin only was used primarily in cases with ruptured membranes and a mature cervix.

### 2.1. Statistics

One-way ANOVA (Analysis of Variance) was used to compare mean values, and the Chi-square test to compare the proportions, between the different methods of induction.

In this study the association between the frequency of CS (primary outcome) and method of induction was analysed, and logistic regression was used to adjust this association with respect to other risk factors for CS. We adjusted for risk factors such as maternal age (<30 years or >30 years), parity (primi- or multiparos), gestational age (<41 + 0 w or ≥ 41 + 0 w), years of induction (2012-2013 or 2009-2010), indication for induction (Premature Rupture of Membrane (PROM), postdate (≥41 w), maternal, fetal, and nonmedical), BS (≤5 or >5), and method of induction (misoprostol, dinoproston^*∗*^ (Minprostin), dinoproston^*∗∗*^ (Propess), amniotomy, balloon, or oxytocin). First, we calculated the crude (unadjusted) associations of each risk factor and CS. Second, we used multivariable models to study the adjusted associations with respect to the risk factors above. Finally, to study whether the method of induction differed in any subgroup with respect to the levels of risk factors, we added an interaction to the adjusted model between the method of induction and each of the risk factors, one at a time. The Hosmer and Lemeshow test and Goodness of Fit test were used. The associations are presented as odds ratios (OR) with 95% confidence intervals (CI). A *p* value < 0.05 was considered statistically significant. Statistical analyses were performed using SPSS 20.0 (SPSS Inc., Chicago, IL). The study was approved by the regional ethics committee (Karolinska Institute, file record: 2014/757-31/2).

## 3. Results

**Figure 1:**
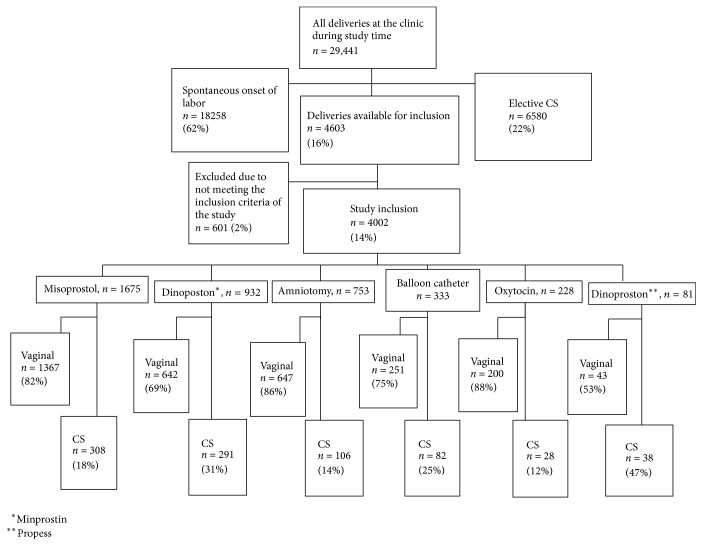
Flow chart of study design.

During the four years of the study, 29441 women were delivered at the clinic. 16% (*n* = 4603) of them were induced to labor and 87% (4002/4603) of them met the criteria for inclusion in the study (Figure 1).

Table 1 describes baseline data from all the induced deliveries with respect to the method of induction. Differences between women included were found according to maternal age, parity, indication for induction, and gestational age. Newborns in the groups of dinoproston^*∗*^ (Minprostin) and amniotomy were older (284 days), and the youngest ones were found in the group where oxytocin was used solely (275 days, *p* < 0.001) (Table 1). The most common indication for induction overall was postdated pregnancies (≥41 w of gestation, 27.5%), followed by PROM (22%), nonmedical indication (19.5%), maternal reasons (17%), or fetal reasons (14%) (Table 2).

During 2009-2010, the number of inductions was 1898. First-line method of induction during that period was dinoproston^*∗*^ (Minprostin, 48%), followed by amniotomy (25%), balloon catheter (15.6%), and dinoproston^*∗∗*^ (Propess, 3.2%). During 2012-2013, the number of inductions was 2104, and the first-line method of induction was changed to misoprostol (80%) followed by amniotomy (13.5%), balloon catheter (1.6%), and dinoproston^*∗*^ (Minprostin, 0.5%) (Table 1).

Induction of labor in an immature cervix strives to reach amniotomy. Amniotomy itself is therefore not considered as an additional method of induction. In 4.7% (79/1675) deliveries in the misoprostol group, in 17.3% (161/932) of deliveries in the dinoproston^*∗*^ (Minprostin) group, in 12.3% (10/81) of deliveries in the dinoproston^*∗∗*^ (Propess) group, and in 0.3% (1/333) in the balloon group, an additional method was used after the primary method of induction.

Cervical ripening at the start of the induction differed among the groups. 2825 (71%) of the women had a BS ≤ 5. In the groups where some kind of prostaglandin had been used it was more common with BS ≤ 5 (misoprostol 90%, dinoproston^*∗*^ (Minprostin, 94%), and dinoproston^*∗∗*^ (Propess, 94%)), compared to the other groups (balloon catheter 63%, amniotomy 15%, and oxytocin 17%) (Table 2).

The time from first action until delivery differed according to outcome of delivery. In deliveries where a CS was performed, the length of delivery was in mean 22 h (0.6–104 h). In the group of vaginal deliveries, the mean time from first action until delivery was 15.5 h. (1.2–88 h). In deliveries with a low BS (≤3) from the start, no differences in length of delivery were shown when misoprostol or dinoproston^*∗*^ (Minprostin) was compared (Table 2). Vaginal Delivery in 24 hours (VD24) occurred in 65% of the induced deliveries. VD24 occurred in 60% of the women induced with misoprostol and in 52% with dinoproston^*∗*^ (Minprostin). The lowest rate of VD24 was found in the dinoproston^*∗∗*^ (Propess) group with 32% (*p* = 0.014) (Table 2).

The total number of CS in the study cohort was 853 (21.3%). The frequency of multiparous women with a previous CS in this study is 231/1639 (14.1%). Among deliveries with an immature cervix at the start (BS ≤ 5) the lowest frequency of CS was seen in the group where the solution of misoprostol was administrated (18%), and the highest frequency was found in the group of dinoproston^*∗∗*^ (Propess, 47%), followed by the group of dinoproston^*∗*^ (Minprostin, 31%) (Table 2). The CS rate was 628/2363 (27%) among primiparous women compared with 225/1638 (14%) among multiparous. The lowest rate of CS overall, for both primi- and multiparous women with an immature cervix, was found in the group where misoprostol had been administrated orally (23 versus 11%) (Table 3).

Among the newborns, 0.7% (*n* = 28) had an Apgar score <7 at 5 minutes. PH (artery) in cord blood <7.10 was present in 3.2%, (*n* = 127) with the highest incidence in the amniotomy group (4%, *n* = 29/753, *p* = 0.1).

All well-known risk factors previously associated with induced deliveries ending in a CS (maternal age > 30, primiparity, and gestational age ≥ 41 w) [7] also had a positive association in this study (*p* < 0.001) (Table 4).

When the indications for labor induction were studied such as postdate pregnancy (OR 1.7; 95% CI 1.3–2.2), maternal reason (OR 1.8; 95% CI 1.4–2.4), or fetal reason (OR 1.5; 95% CI 1.1–2.0), all were associated with CS except “nonmedical reason” (Table 4).

Induced deliveries with an immature cervix (BS ≤ 5) were associated with an increased risk of ending in a CS (OR 1.7; 95% CI 1.5–2.1). After adjustment for the other risk factors, the result was no longer significant (aOR 1.2; 95% CI 0.94–1.6). When studying the six methods of induction used, all of them except the amniotomy and oxytocin groups were associated with CS in the crude analysis, but not after adjustment for the other risk factors except for dinoproston^*∗∗*^ (Propess) which had a 2,9 increased OR for CS (aOR = 2.9 (1.6–5.2)) (Table 4). No significant interactions were found (*p* values between 0.17 and 0.95). The Hosmer and Lemeshow test and Goodness of Fit test were equal to *p* = 0,018 for the adjusted model (*n* = 4001).

## 4. Discussion

Main findings in this study of 4002 induced deliveries were that an orally administrated solution of misoprostol is an effective method of induction. Orally given solution of misoprostol has a high success rate and a low proportion of CS. An oral solution of misoprostol works safely and effectively when labor needs to be induced.

Our main findings are consistent with earlier studies, such as the study published by Aghideh et al. [16], and in the Cochrane review recently published by Alfirevic et al. [2]. Trials with oral administration of misoprostol were compared with placebo and other methods for induction. Compared to placebo, women with orally administrated misoprostol were shown to have a higher rate of VD24. Further, women who received oral misoprostol had a lower rate of CS compared to women who received vaginal dinoproston or placebo [2, 17]. The Cochrane review concluded that an oral administration of 20–25 micrograms of misoprostol is recommended due to better performance and a high level of acceptance among the delivering women. The dosage does not lead to a higher frequency of hyperstimulation of the uterus or affected CTG compared with other prostaglandins. Similarities with our results are noted. The acceptance among the included women was high even in our study and no higher frequency of poor fetal outcome or other complications was found. A low rate of CS in the misoprostol group was presented (18%), and this is particularly notable in the subgroup of primiparous women where the rate of CS normally is known as high (23% in this study).

In other studies, published on orally administered misoprostol, the preparation is usually given either buccally or sublingually [5, 12, 14, 17–22], and not like in this present study as a solution. Misoprostol tablets are available in 200 micrograms and dividing them into eight pieces could pose a risk for poor dosing accuracy. This problem can be avoided by preparing a solution to drink, and the compliance will be better [13]. The method to solve a tablet in water as a mixture and administrate it as a solution to drink every second hour has been developed in the women's clinic of Sodersjukhuset during recent years and refined to become an effective method of induction. The clinical experience of using this method is that the onset of labor, induced with an oral solution of misoprostol, has many similarities with a spontaneously onset of labor. The way misoprostol is used at Sodersjukhuset is in our opinion unique. Our data indicate that this administration of misoprostol seems to be preferable compared to other ways of administration, especially for avoiding complications, hyperstimulations, and a high rate of CS.

In the analysis of the different risk factors for CS in induced deliveries, [17, 23], it seems like these factors have a high importance for the outcome of delivery, perhaps more than the method itself that has been used. The conclusion must be that well-known risk factors of CS in induced deliveries have a big impact of delivery outcome, regardless the way of induction. On the other hand, when conditions are the same it seems like orally given misoprostol is well comparable with other methods of induction [24]. We conclude that when conditions are the same, use of an oral solution of misoprostol the way it has been used at Sodersjukhuset, at an immature cervix, gives a lower rate of CS compared with dinoproston and balloon, without affecting duration of delivery or the maternal or fetal outcome. It is also important to notice that the acceptance among the included women is high and according to our clinical experience they seem to be more satisfied with this method of induction.

We believe that oral given misoprostol should be recommended as the primary method of induction especially as the different levels of BS appear to be of minor importance according to our results.

*Strengths and Limitations in This Study.* Although misoprostol is recommended for labor induction by the WHO, the use of misoprostol is currently debated in the media, within the profession, and among pregnant women. Misoprostol is still commonly used off label. Our study results are of importance not only for obstetrical care in Sweden or other high resource settings but also in countries with limited resources. Finding an effective and safe method of induction, a method that can be used safely and also globally must be considered of the highest importance. No large studies where a solution of misoprostol has been given orally like the way it has been given in this project are available. The greatest strength of our study is the large number of included women, 1675, who were induced with this special mixture. This makes this study one of largest projects presented in this research filed. All data was checked in the women's records after delivery. This will increase the reliability compared to other big registry studies of this subject. Further prospective studies should be planned and performed to refine the use of the method.

Some limitations of this study should be mentioned. Sodersjukhuset is a large city hospital in Stockholm and the women included in the study were older and probably better educated than the national average in Sweden. This might have some influence on the findings. Another limitation of our project is that this in fact is a retrospective, nonrandomised controlled study.

## 5. Conclusions

An orally administrated solution of misoprostol is a good method of induction. Orally given misoprostol is preferred by the delivering women and has a high rate of success and a low proportion of CS. Administering a prepared solution of misoprostol to drink, compared with other pharmaceutical forms, facilitates correct dosage and improves compliance. If the time of labor can be shortened, the frequency of CS decreased, and the proportion of healthy mothers and children increased this will lead to safer and better obstetrical care and probably even to a shorter length of stay in hospital, which is interesting also from a socioeconomic perspective.

## Figures and Tables

**Table 1:** Maternal and fetal background data, presented per method of induction. Data are presented as numbers (%) or mean (SD). *n* = 4002.

Maternal	Misoprostol (*n* = 1675)	Dinoproston^*∗*^ (*n* = 932)	Amniotomy (*n* = 753)	Balloon catheter (*n* = 333)	Oxytocin (*n* = 228)	Dinoproston^*∗∗*^ (*n* = 81)	*p* value^*∗∗∗*^
Age (year)	33.9 (5.0)	33.3 (5.0)	33.1 (5.0)	32.7 (5.0)	33.1 (4.9)	32.7 (5.4)	<0.001^*∗∗∗*^
Parity (primiparous%)	1039 (62)	574 (62)	348 (46)	205 (62)	147 (64)	51 (63)	<0.001^*∗∗∗*^
BMI (kg/m^2^)	24.5 (4.4)	24.7 (4.5)	24.4 (4.7)	24.0 (4.0)	24.1 (4.3)	24.0 (5.1)	0.13
Maternal reason for induction (*n* = 690)							<0.001^*∗∗∗*^
PE^*◧*^ (%, *n* = 247)	158 (9)	46 (5)	27 (4)	5 (1.5)	3 (1.5)	8 (10)	
ICP^*ɤ*^	41 (2.5)	11 (1)	10 (1.5)	9 (2.5)	1 (0.5)	1 (1)	
(%, *n* = 73)							
Diabetes (% *n* = 43)	24	11	7	0	0	1	
	(1.5)	(1)	(1)			(1)	
HT° (%, *n* = 37)	19	9	7	2	0	0	
	(1)	(1)	(1)	(0.5)			
Others (%, *n* = 290)	42 (2.5)	132 (14)	60 (8)	37 (11)	4 (2)	15 (19)	
Fetal reason for induction (*n* = 555)							<0.001^*∗∗∗*^
Oligohydramnion (%, *n* = 200)	158	46	30	14	0	6	
	(9)	(5)	(4)	(4)		(7)	
DFM^∧^ (%, *n* = 143)	41	11	31	8	0	1	
	(2.5)	(1)	(4)	(2)		(1)	
IUGR^∧∧^ (%, *n* = 97)	24 (1.5)	11 (1)	10 (1.5)	2 (0.5)	3 (1)	11 (14)	
CTG^∧∧∧^ (%, *n* = 56)	19 (1)	9 (1)	19 (2.5)	1 (0.5)	0	3 (4)	
Others (%, *n* = 59)	42 (2.5)	132 (14)	15 (2)	7 (2)	1 (0.5)	14 (17)	
Gestational age (days)	282 (12)	284 (13)	284 (11)	283 (12)	275 (12)	276 (13)	<0.001^*∗∗∗*^
Gender (boys)	921 (55)	504 (54)	414 (55)	173 (52)	130 (57)	40 (49)	0.8
Years of induction							
2009-2010	0	922	471	300	144	61	
2012-2013	1675	10	282	33	84	20	

^*∗*^Minprostin, ^*∗∗*^Propess, and ^*∗∗∗*^*p* values < 0.05 were considered as statistically significant. ^*◧*^Preeclampsia. ^*ɤ*^Intrahepatic  cholestasis. °Hypertension. ^∧^Decreased fetal movements. ^∧∧^Intrauterine growth retardation; ^∧∧∧^suspect abnormal CTG.

**Table 2:** Bishop score, indications for induction, and labor outcome data, presented per *method of induction*. Data are presented as numbers (%) or mean (SD).

	Misoprostol (*n* = 1675)	Dinoproston^*∗*^ (*n* = 932)	Amniotomy (*n* = 753)	Balloon catheter (*n* = 333)	Oxytocin (*n* = 228)	Dinoproston^*∗∗*^ (*n* = 81)	*p* value^*∗∗∗*^
Bishop score (0–10)	3 (2)	3 (1)	7 (1)	5 (1)	7 (1)	3 (2)	<0.001^*∗∗∗*^
Indications for induction							
Postdate pregnancies^∧^ (%, *n* = 1093)	473 (28.0)	314 (34.0)	208 (28.0)	87 (26.0)	7 (3.0)	4 (5.0)	<0.001^*∗∗∗*^
PROM^∧∧^ (%, *n* = 886)	374 (22.5)	168 (18.0)	52 (7.0)	92 (28.0)	193 (85.0)	7 (9.0)	
Maternal reason (%, *n* = 690)	284 (17)	209 (22.0)	111 (15.0)	53 (16.0)	8 (3.5)	25 (31.0)	
Fetal reason (%, *n* = 555)	292 (17.5)	90 (10.0)	105 (14.0)	29 (9.0)	4 (2.0)	35 (43.0)	
Nonmedical reason (%, *n* = 778)	252 (15.0)	151 (16.0)	277 (37.0)	72 (22.0)	16 (7.0)	10 (12.0)	
Time from induction to delivery (h)							<0.001^*∗∗∗*^
(i) Vaginal	19	19	8	11	9	2	
(ii) CS	26	25	12	14	13	17	
Vaginal delivery in 24 h (VD24, %)	1010 (60.0)	487 (52.0)	642 (85.0)	249 (75.0)	195 (86.0)	26 (32.0)	0.014^*∗∗∗*^
Way of delivery (%)							<0.001^*∗∗∗*^
(i) Vaginal	1367 (82.0)	642 (69.0)	647 (86.0)	251 (75.0)	28 (12.0)	43 (53.0)	
(ii) CS	308 (18.0)	291 (31.0)	106 (14.0)	82 (25.0)	200 (88.0)	38 (47.0)	
PPH > 1500 ml^∧∧∧^ (*n* = 182, %)	81 (5.0)	44 (5.0)	29 (4.0)	18 (5.5)	7 (3.0)	3 (4.0)	0.4
Apgar < 7,5′ (*n* = 29, %)	16	8	2	3	0	0	0.04^*∗∗∗*^
	(1.0)	(1.0)	(0.5)	(1.0)			
pH < 7.10 (*n* = 127, %)	59	30	29	4	5	0	<0.001^*∗∗∗*^
	(3.5)	(3.0)	(4.0)	(1.0)	(2.0)		

^*∗*^Minprostin , ^*∗∗*^Propess, and ^*∗∗∗*^*p* values < 0.05 were considered statistically significant. ^∧^>41 w. ^∧∧^Prerupture of the membranes. ^∧∧∧^Postpartum hemorrhage.

**Table 3:** Bishop score, indications for induction, and labor outcome data, presented per *method of induction *and separated for *primi- and multiparos *women (P/M). Data are presented as numbers (%) or mean (SD).

	Misoprostol *n* = 1040/635	Dinoproston^*∗*^ *n* = 573/359	Amniotomy *n* = 349/404	Balloon catheter *n* = 205/128	Oxytocin *n* = 147/81	Dinoproston^*∗∗*^ *n* = 51/30	*p* value^*∗∗∗*^
Bishop score (0–10)	3/3	3/3	7/7	5/5	7/6	3/3	<0.01/0.01^*∗∗∗*^
Time from induction to delivery (h)							<0.01/0.01^*∗∗∗*^
(i) Vaginal	22/16	20/16	10/6	14/10	10/7	16/17	
(ii) CS	27/22	25/26	12/11	12/14	13/14	22/21	
Postdate pregnancies^*◧*^ (*n* = 713/38, %)	339/134 (32.5/21)	217/97 (38/27)	102/106 (29/26)	49/38 (24/30)	3/4 (2/5)	3/1 (6/3.5)	
Indications for induction							
PROM^*ɤ*^ (*n* = 609/27, %)	272/102 (26/16)	102/66 (18/18.5)	37/15 (11/4)	62/30 (30/23)	130/63 (88.5/78)	6/1 (12/3.5)	
Maternal Reason (*n* = 412/27, %)	183/101 (17.5/16)	125/84 (22/23.5)	49/62 (14/15)	34/19 (17/15)	5/3 (3.5/4)	16/9 (31/30)	
Fetal reason (*n* = 307/24, %)	165/127 (16/20)	55/35 (10/10)	45/60 (13/15)	16/13 (8/10)	3/1 (2/1)	23/12 (45/40)	
Nonmedical reason (*n* = 324/45, %)	81/171 (8/27)	74/77 (13/21)	116/161 (33/40)	44/28 (21/22)	6/10 (4/12)	3/7 (6/23)	
Way of delivery							<0.01/0.01^*∗∗∗*^
Vaginal (%)	801/566 (77/89)	368/274 (64/76)	275/372 (79/92)	145/106 (71/83)	122/78 (83/96)	25/18 (49/60)	
CS (%)	238/70 (23/11)	206/85 (36/24)	73/33 (21/8)	60/22 (29/17)	25/3 (17/4)	26/12 (51/40)	
PPH^∧^ > 1500 ml (*n* = 124/58, %)	61/20 (6/3)	26/16 (5/4)	17/12 (5/3)	14/4 (7/3)	5/4 (3/5)	1/2 (2/7)	*p* = 0,2
Apgar < 7,5′ (*n* = 19/9, %)	13/2 (1/0.3)	3/5 (0.5/1)	1/1 (0.3/0.3)	2/1 (1/0.8)	0/0 —	0/0 —	*p* = 0,1
pH < 7.10 (*n* = 94, 42%)	51/13 (5/2)	21/12 (4/3)	16/13 (5/3)	2/3 (1/2)	4/1 (3/1)	0/0 —	*p* = 0.7

^*∗*^Minprostin, ^*∗∗*^Propess, and ^*∗∗∗*^*p* values < 0.05 were considered statistically significant. ^*◧*^>41 w. ^*ɤ*^Prerupture of the membranes. ^∧^Postpartum hemorrhage.

**Table 4:** Associations between possible risk factors and CS. Values are expressed as odds ratio (OR) with corresponding 95% confidence intervals (CI). *N* = 4001 (1 missing).

Risk factors for CS during delivery	CS/total (%)^*∗∗*^	OR unadjusted (95% CI)	OR adjusted (95% CI)
Maternal age			
<30 years	161/856 (19)	Ref.	Ref.
≥30	677/3091 (22)	1.2 (1.0–1.5)^*∗∗∗*^	1.5 (1.2–1.8)^*∗∗∗*^
Parity			
Multiparos	225/1639 (14)	Ref.	Ref.
Primiparous	628/2364 (27)	2.3 (1.9–2.7)^*∗∗∗*^	2.4 (2.0–2.8)^*∗∗∗*^
Gestational age (days)			
<41 + 0	344/2072 (17)	Ref.	Ref.
≥ 41 + 0	485/1824 (27)	1.8 (1.5–2.1)^*∗∗∗*^	1.9 (1.5–2.3)^*∗∗∗*^
Indication for induction			
(i) PROM	147/881 (17)	Ref.	Ref.
(ii) Postdate	269/1090 (25)	1.6 (1.3–2.0)^*∗∗∗*^	1.7 (1.3–2.2)^*∗∗∗*^
(iii) Maternal (>41 w)	197/712 (28)	1.9 (1.5–2.4)^*∗∗∗*^	1.8 (1.4–2.4)^*∗∗∗*^
(iv) Fetal	91/434 (21)	1.3 (1.0–1.8)^*∗∗∗*^	1.5 (1.1–2.0)^*∗∗∗*^
(v) Nonmedical	148/885 (17)	1.0 (0.8–1.3)	1.3 (0.96–1.7)
Years of induction			
(i) 2012-2013	358/2085 (17)	Ref	Ref
(ii) 2009-2010	495/1918 (26)	1.7 (1.4–2.0)^*∗∗∗*^	1.5 (1.0–2.0)^*∗∗∗*^
Bishop score			
>5	167 (1123) (15)	Ref.	Ref.
≤5	661/2780 (24)	1.7 (1.5–2.1)^*∗∗∗*^	1.2 (0.94–1.6)
Method of induction			
(i) Misoprostol	308/1675 (18)	Ref.	Ref.
(ii) Dinoproston^*∗*^	291/933 (31)	2.0 (1.7–2.4)^*∗*^	1.4 (0.96–2.1)
(iii) Amniotomy	106/753 (14)	0.7 (0.6–0.9)^*∗*^	0.8 (0.5–1.2)
(iv) Balloon catheter	82/333 (25)	1.4 (1.1–1.9)^*∗*^	1.2 (0.8–1.9)
(v) Oxytocin	28/228 (12)	0.6 (0.4–0.9)^*∗*^	0.8 (0.5–1.3)
(vi) Dinoproston^*∗∗*^	38/81 (47)	3.9 (2.5–6.2)^*∗*^	2.9 (1.6–5.2)^*∗*^

^*∗*^Minprostin, ^*∗∗*^Propess, and ^*∗∗∗*^*p* values < 0.05 were considered as statistically significant.
